# Supplementary material for: Simultaneous impairment of neuronal and metabolic function of mutated gephyrin in a patient with epileptic encephalopathy
Source: EMBO Mol Med. 2015 Nov 27;7(12):1580–94. doi: 10.15252/emmm.201505323 (PMC4693503; doi:10.15252/emmm.201505323)
Supplement: Supplementary file 1 — Expanded View Figures PDF [file EMMM-7-1580-s001.pdf]

Expanded View Figures

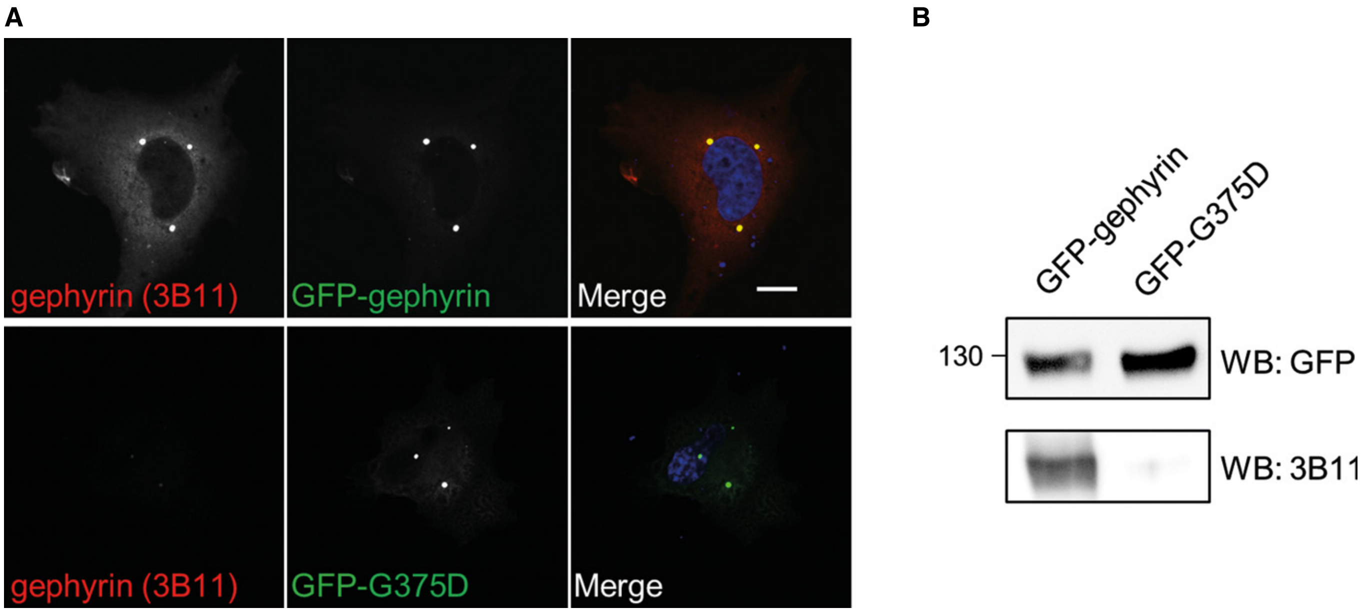

Figure EV1. Monoclonal 3B11 antibody does not bind to gephyrin-G375D.

A COS7 cells transfected with GFP-tagged gephyrin or G375D and immunostained with the monoclonal 3B11 antibody (red). Scale bar, 10 μm.

B HEK293 cells transfected as in (A) and immunoblotted with GFP-specific or 3B11 antibodies. Note that 3B11 does not detect G375D in any of these assays.

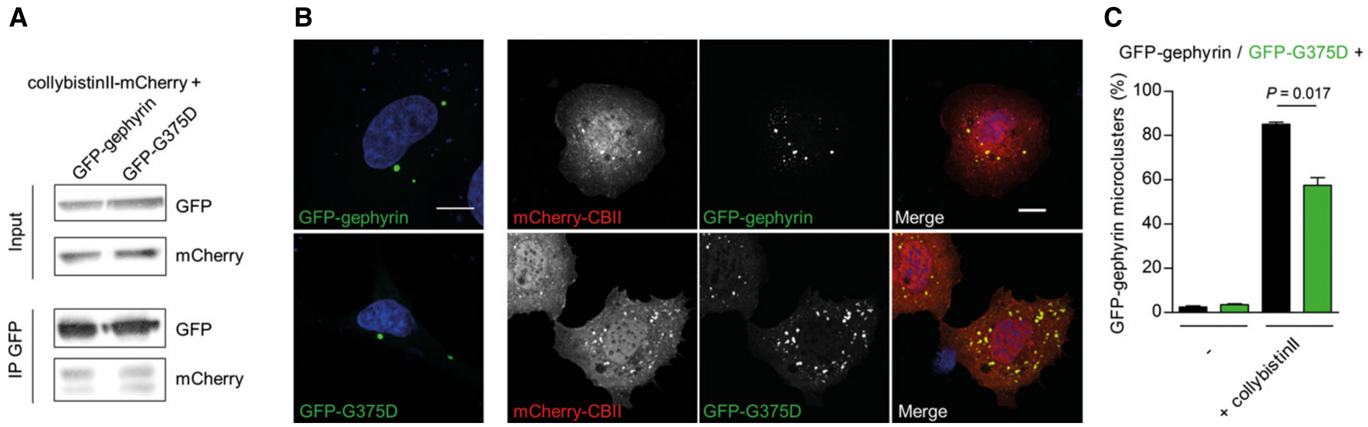

Figure EV2. Gephyrin-G375D binds to collybistin II leading to the formation of submembranous microclusters in non-neuronal cells.

A Lysates and co-immunoprecipitation of HEK293 cell lysates co-expressing GFP-tagged gephyrins and mCherry-tagged collybistin II. GFP-specific antibodies were used for the immunoprecipitation.

B Images of COS7 cells (co)-expressing GFP-gephyrin or GFP-G375D with mCherry-tagged collybistin II (CBII). Note that gephyrin forms large cytoplasmic aggregates ('blobs'). CBII induces the formation of gephyrin microclusters at the plasma membrane and co-localizes with the microclusters in both gephyrin variants. Scale bars, 10 μm.

C Quantification of GFP-gephyrin and GFP-G375D microclusters in (co)-transfected COS7 cells as indicated (microcluster, submembranous localization; 100 cells from two independent transfections were analyzed). Gephyrin in remaining cells either formed blobs or was diffusively distributed. Results are expressed as mean ± SEM and were analyzed using *t*-test.

© 2015 The Authors

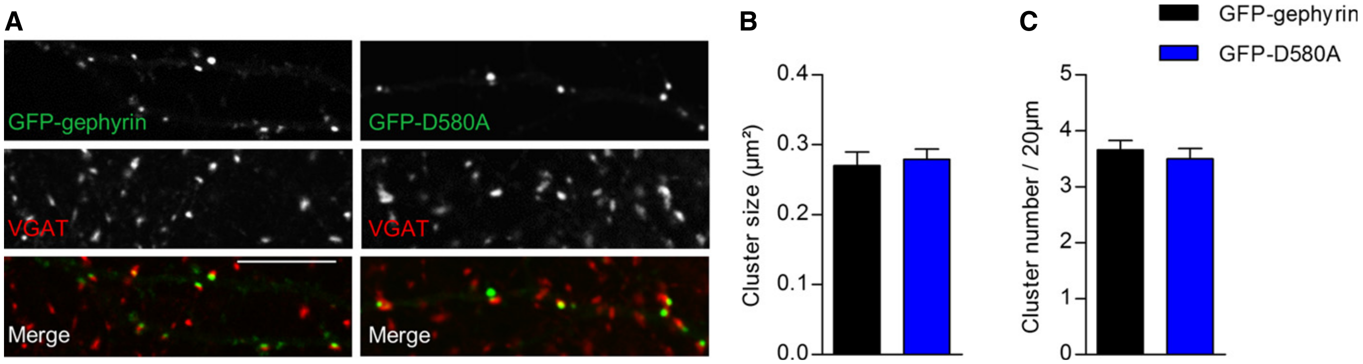

Figure EV3. Gephyrin-D580A forms regular postsynaptic clusters in primary neurons.

A Representative dendritic segments of neurons expressing GFP-gephyrin or GFP-D580A immunostained with VGAT (red). Note that both gephyrin variants co-localize with VGAT puncta. Scale bar, 10 µm.

B, C Quantification of GFP-gephyrin cluster size (B) and quantity (C) in hippocampal neurons expressing GFP-gephyrin or GFP-D580A. 19 GFP-gephyrin and 17 GFP-D580A neurons from three independent cultures were used for quantifications. Results are expressed as mean ± SEM.

 © 2015 The Authors
